# Epidemiology survey of infectious diseases in North Korean travelers, 2015–2017

**DOI:** 10.1186/s12879-018-3664-x

**Published:** 2019-01-05

**Authors:** Pengyu Han, Yanxia Teng, Xiuxin Bi, Jinge Li, Dianxing Sun

**Affiliations:** 1 Department of Infectious Diseases, Bethune International Peace Hospital, NO.398, Zhongshanxi Road, Shijiazhuang, Hebei Prov 050082 People’s Republic of China; 2 Dandong Customs, Dandong, China; 3 Dadonggang Customs, Donggang, China; 4 Dandong International Travel Healthcare Center, Dandong, China

**Keywords:** DPRK, Hepatitis B virus, Tuberculosis, Syphilis, Malaria, Port

## Abstract

**Background:**

Up until now, there are limited studies available on the epidemiology of infectious diseases in Democratic People’s Republic of Korea (DPRK, North Korea). However, different types of infectious diseases have been found in North Korean travelers at Dandong port. Entry surveillance data of those North Korean travelers may provide some insight into the probable epidemiology of some infectious diseases in DPRK.

**Methods:**

We actively analyzed the medical test result of North Korean travelers entering China through Dandong port. Detection of hepatitis B virus (HBV), tuberculosis (TB), syphilis and malaria was made by specific laboratory tests according to the national technical guidelines. Infectious diseases surveillance data for 2015–17 was analyzed and compared among subgroups.

**Results:**

Between Jan 1, 2014, and Dec 31, 2016, 557 cases of infectious diseases were identified among 18,494 North Korean travelers, HBV active infection (466 cases), active TB infection (33 cases), current active syphilis infection (57) cases, *Plasmodium falciparum (P.falciparum)* malaria infection (1 case). The incidence of HBV, TB and syphilis in North Korean travelers was high. Incidence of TB increased from 11.7256 (1/10,000) in 2015 to 28.2738 (1/10,000) in 2017. HBV immunization rate in in North Korean travelers was relatively high in 0–10 age group.

**Conclusion:**

This report is the first to characterize the profile of infectious diseases among arriving North Korean travelers in mainland China. Our findings suggest high incidence of HBV, TB and syphilis among North Korean travelers. The screening for TB in North Korean workers should be strengthened in order to prevent infections imported into China.

## Background

DPRK rarely reports its domestic health status to the international world. Up until now, only limited researches were conducted to investigate the prevalence of infectious disease in DPRK. However, researches from the World Health Organization (WHO) and other international groups show that TB, HBV, malaria and other communicable diseases are still rampant in DPRK [1–4]. It is estimated that there is a high prevalence of HBV infection in DPRK due to poor vaccination status and lack of antiviral treatment [5]. In 2003, a filed survey conducted by WHO showed that the prevalence of HBV was 4.5% in DPRK, while other studies showed a higher prevalence of 20% in some rural areas [5–7]. Data from WHO indicates the prevalence of TB in DPRK was 399/100,000 in 2011, 345/100,000 in 2012 and 442/100,000 in 2014 [8–11]. In 2016, DPRK launched its first national field TB prevalence survey which showed a prevalence of 634/100,000 [12]. With the help of global founds, North Korea has developed their own TB treatment program [13], but exactly how these found were used remains largely unknown [14]. DPKR has suffered from *Plasmodium vivax* (*P.vivax)* malaria epidemic several times in history [15]. Successful experience of using primaquine preventive treatment (MPPT) to treat malaria in China has encouraged DPRK to start implementing their own MPPT program which significantly reduced the prevalence of *P.vivax* malaria in DPRK. As a result, reported cases of malaria infection in DPRK dropped from 296,540 in 2001 to 5113 in 2016 [15].

Dandong is the largest border city in China and Dandong port is the largest land port connecting China and DPRK. Data from the former China Inspection and Quarantine Bureau (CIQ) suggests that there are about 50,000 North Korean travelers entering China through Dandong port each year. The objective of the present study was to investigate the incidence of hepatitis, syphilis, malaria and tuberculosis in those North Korean travelers.

## Methods

According to core capacity requirements for surveillance and response in WHO’s International Health Regulations and the Frontier Health and Quarantine Law of China, all foreigners intend to work or live in China must undergo medical examinations which include tests for HBV, Syphilis and tuberculosis [16, 17]. As for travelers who have been to epidemic areas, test for related diseases such as malaria and yellow fever are advised. In routine examination, internal medicine check, electrocardiogram examination, abdominal B-ultrasound and chest X-ray examination were performed. In Laboratory tests, Elisa and fluorescence quantitative PCR, toluidine red unheated serum test (TRUST) and treponema pallidum antibody agglutination test (TPPA) were performed at Liaoning International Travel Healthcare Center (ITHC); Sputum smear and mycobacterium tuberculosis culture were performed to detect tuberculosis at Shenyang International Travel Healthcare Center.Epidemiology filed survey was conducted in a Chinese factory where 5 cases of imported pulmonary TB were found. Factory workers were asked to complete a questionnaire with questions about demographics, pulmonary TB awareness and TB exposure history. A follow-up investigation of the sanitary measures adopted in the factory was performed.

## Results

Between Jan 1, 2015, and Dec 31, 2017, 18,494 North Korean nationals were examined for infectious diseases, 4521(24.45%) were male and 13,973 (75.55%) were female; 557 cases of infectious diseases were detected in participants: active HBV infection (466 cases), current active syphilis infection (57 cases), active TB infection (33 cases), P.vivax malaria infection (1 case). Participants were divided into 7 age groups, 20–30 age group had the highest incidence of infectious disease, 134.0975(1/10,000) (248 cases).

The number of cases imported varied greatly by reasons for travel, 16,074 (86.91%) were labor workers, 1354(7.32%) were on business trip; 544 (2.94%) were foreign marriages; 473(2.56%) were students; incidence of active HBV active infection was highest in business trip group (420.9749, 1/10,000) followed by Marriage (257.3529, 1/10,000) and export labor workers (243.8721, 1/10,000); incidence of syphilis and TB infection were highest in labor workers; incidence of syphilis and TB infection in North Korean nationals had increased from 2015 to 2017; incidence of active HBV infection was highest in 2016 and dropped in 2017 (Table 1).

**Table 1:** Demography distribution of infectious diseases in North Korean travelers

Subject	Overall	HBV active infection		Current active syphilis		TB	
	N (%)	Case	Prevalence(1/10,000)	Case	Prevalence(1/10,000)	Case	Prevalence(1/10,000)
Gender							
Male	4521 (24.45)	264	583.9416	15	33.1785	0	0.0000
Female	13,973 (75.55)	202	144.5645	42	30.0580	33	23.6170
Age							
0–10	15 (0.08)	0	0.0000	0	0.0000	0	0.0000
11–20	2937 (15.88)	18	60.9756	1	3.3875	2	6.7751
21–30	9436 (51.02)	195	206.6554	25	26.4943	26	27.5540
31–40	2674 (14.46)	94	351.5333	20	74.7943	3	11.2191
41–50	2387 (12.91)	101	423.1253	7	29.3255	2	8.3787
51–60	982 (5.31)	58	590.6314	4	40.7332	0	0.0000
≥61	63 (0.34)	0	0.0000	0	0.0000	0	0.0000
Reason for travel							
Labor Worker	16,074 (86.91)	392	243.8721	56	34.8389	32	19.9079
Business Trip	1354 (7.32)	57	420.9749	1	7.3855	0	0.0000
Student	473 (2.56)	3	63.4249	0	0.0000	0	0.0000
Traveler	48 (0.26)	0	0.0000	0	0.0000	0	0.0000
Marriage	544 (2.94)	14	257.3529	0	0.0000	1	18.3824
Others	1 (0.01)	0	0.0000	0	0.0000	0	0.0000
Year							
2015	5117 (27.67)	89	173.9300	13	25.4055	6	11.7256
2016	6657 (35.60)	255	383.0554	17	25.5370	8	12.0174
2017	6720 (36.34)	122	181.5476	27	40.5588	19	28.2738

HBV infection and immunization status were analyzed in different age groups, 0–10 age group had the highest immunization rate (46.67%), 51–60 age group had the highest HBV active infection rate (5.91%), 41–60 age group had the highest HBV past infection rate (0.92%), 11–20 age group had the highest HBV negative rate (98.71%) (Table 2).

**Table 2:** HBV infection status in North Korean travelers

Age	HBV immunization		HBV active infection		HBV past infection		HBV negative	
(year)	(N)	(%)	(N)	(%)	(N)	(%)	(N)	(%)
0–10	7	46.67	0	0.00	0	0.00	8	53.33
11–20	16	0.54	18	0.61	4	0.13	2899	98.71
21–30	51	0.54	195	2.07	15	0.16	9175	97.23
31–40	3	0.12	94	3.52	14	0.52	2563	95.85
41–50	15	0.63	101	4.23	22	0.92	2249	94.22
51–60	17	1.73	58	5.91	9	0.92	898	91.44
61–70	3	4.84	0	0.00	4	6.45	55	88.71

Gender and age distribution of syphilis were described, 42 cases with current activity of syphilis infection were female and 12 cases were male; in female group, incidence of past syphilis infection was highest in 41–50 age group (145.2785, 1/10,000) followed by 31–40 age group (49.2368, 1/10,000), incidence of current active syphilis was highest in 31–40 age group (64.0079, 1/10,000) followed by 41–50 age group (60.5327, 1/10,000) and 21–30 age group (27.2997, 1/10,000); in male group, incidence of past syphilis infection was highest in 41–50 age group (32.0307, 1/10,000) followed by 31–40 age group (31.1042, 1/10,000), incidence of current active syphilis was highest in 31–40 age group (108.8647, 1/10,000) followed by 21–30 age group (59.3472, 1/10,000) and 41–50 age group (12.8123, 1/10,000) (Table 3).

**Table 3:** Syphilis infection status in North Korean travelers

Age	Overall				Past syphilis				Current active syphilis			
(year)	Male		Female		Male		Female		Male		Female	
	(N)	(%)	(N	(%)	(N)	(1/10,000)	(N)	(1/10,000)	(N)	(1/10,000)	(N)	(1/10,000)
0–10	9	60.00	6	40.00	0	0.0000	0	0.0000	0	0.0000	0	0.0000
11–20	611	20.80	2326	70.20	0	0.0000	0	0.0000	0	0.0000	1	4.2992
21–30	1011	10.71	8425	89.29	0	0.0000	3	3.5068	6	59.3472	23	27.2997
31–40	643	24.05	2031	75.95	2	31.1042	10	49.2368	7	108.8647	13	64.0079
41–50	1561	65.40	826	34.60	5	32.0307	12	145.2785	2	12.8123	5	60.5327

In Dec, 2017, 5 cases of active pulmonary TB infection were diagnosed in a group of North Korean female workers entering China through Dandong port. All these workers were destined to work in a garment processing factory in Dandong, 5 confirmed TB cases were young females aged 20–30. Questionnaires were sent to patients’ co-workers and factory managers, only 58 valid questionnaires were collected, 42 were signed by DPRK workers and 16 were signed by factory managers. The questionnaires showed that only 9 (21.43%) workers knew the basic knowledge about TB is and only 3 (9.52%) North Korean workers knew how to prevent TB from transmitting. Those 5 workers were sent back to North Korea right after the diagnosis. Sanitation measures such as disinfection spry and propaganda of TB awareness were performed in this factory by local Central Disease Control (CDC) to prevent further TB transmission.

In Sep, 2015, 1 case of *P.falciparum* malaria infection was found in a North Korean worker who took an international train going back to DPRK through Dandong port. Epidemiology survey revealed that this patient worked in Congo for 1 year and had malaria patient exposure history during his stay in Congo. Unfortunately, the patient went back to DPRK before the test result came out.

## Discussion

To our knowledge, ours is the first study of infectious diseases among North Korean travelers arriving in mainland China. Our findings, which are based on surveillance data, are helpful for providing some insight into the probable epidemiology of some infectious diseases in DPRK and raising public health awareness about the potential risk of imported infections.

In this study, we found that both case numbers and incidence of TB and Syphilis infection had increased over time among North Korean travelers arriving in mainland China. Even though the incidence of HBV active infection in North Korean travelers was still much higher than Chinese national level, both case numbers and incidence of HBV infection in North Korean nationals decreased from 2016 to 2017. Incidence of HBV active infection increased with age while number of HBV negative case decreased with age. We also noticed a high percentage of HBV immunization in North Korean children which may be a result of the national HBV vaccination program applied in North Korea. In recent years, with the help of international medical aid, DPRK has started to vaccinate newborns against HBV nationwide and 7 million children were vaccinated against HBV from 2010 to 2012 with a coverage of 99.23% [5]. We also found that older North Koreans had relatively high HBV immunization rate, the reason for this phenomenon may be that most older travelers are financially well off and have more access to medical resources.

Sexually transmitted disease (STD) is a taboo subject in DPRK. Up until now, there is no data available regards the prevalence of syphilis in DPRK. However, increasing numbers of syphilis case in our study suggest there is a possible high prevalence of syphilis in North Korea. Most syphilis cases were female aged 30–50, we have little knowledge about their sex life, but given the fact that they live in closed social circle, the chance of transmitting syphilis to local Chinese resident is small. No syphilis case was found in North Korean marriage-migrants, which may be a result of the pre-marriage medical screen for couples. In order to better understand the situation of syphilis in North Korean travelers, Dandong Customs will carry out education programs about STD in port areas and offer sanitary supplies and condoms to migrant workers for free.

Socioeconomic factors may account for the uneven distribution of TB cases in different travel groups. North Korean business men and oversea students are often better off economically than labor workers. Most TB cases found in this study were labor workers and not a single TB case was found in business or student group. The number of TB cases in North Korean labor workers found at Dandong port kept increasing for the last 3 years. The majority of North Korean workers in Dandong city are young female working in factories. Those migrant female workers always travel in group with one or two supervisors around and rarely talk to strangers. The living condition for those workers in China is generally poor with only few exceptions for those who work at fancy restaurants. All TB cases in this study were found in female workers in cloth factories, and not a single case was found in those who work at restaurants. Workers in restaurant are mostly come from wealthy families in North Korean and have received better education with better living condition than those who works at factories. All North Korean travelers with active TB infection were sent back to DPRK right after the diagnosis which left us little time to perform more medical test or offer proper treatment.

Up until now, North Korea has never reported a single case of local *P.falciparum* malaria infection. However, imported malaria of export laborers from Africa will increase the chance of local transmission of *P.falciparum* in North Korea. We have also noticed that the average age of North Korean travelers with infectious diseases were getting younger, especially in case of TB infection. Labor workers are the main source of entry North Korean travelers at Dandong port, and have the highest incidence of TB infection.

## Conclusion

Our findings suggest that North Korean travelers,who enter China through Dandong port,have high incidence of TB, HBV and syphilis infection. Active surveillance at China-DPRK port can help with timely diagnosis of travelers with infectious diseases to prevent or at least postpone imported local transmission. In addition to entry surveillance, collaborations between China and international world are needed to help DPRK with their health related issues. We are looking forward to establish a fast and efficient way of sharing infectious diseases outbreak news with DPRK and conduct epidemiology field survey in the future. We are also reaching out for international cooperation to assist DPRK in setting up medical training programs.

## Abbreviations

CDC: Central Disease Control
CIQ: China Inspection and Quarantine Bureau
DPRK: Democratic People’s Republic of Korea
ITHC: International Travel Healthcare Center
TB: Tuberculosis
WHO: World Health Organization
